# Modeling the length distribution of gene conversion tracts in humans from the UK Biobank sequence data

**DOI:** 10.1371/journal.pgen.1011951

**Published:** 2025-11-17

**Authors:** Nobuaki Masaki, Sharon R. Browning

**Affiliations:** Department of Biostatistics, University of Washington, Seattle, Washington, United States of America; University of Melbourne, AUSTRALIA

## Abstract

Non-crossover gene conversion is a type of meiotic recombination characterized by the non-reciprocal transfer of genetic material between homologous chromosomes. Gene conversions are thought to occur within relatively short tracts of DNA. In this study, we propose a statistical method to model the length distribution of gene conversion tracts in humans, using nearly one million gene conversion tracts detected from the UK Biobank whole autosome data. To handle the large number of tracts, we designed a computationally efficient inferential framework. Our method further accounts for regional variation in the density of variant sites and heterozygosity across the genome, which can influence the observed length of gene conversion tracts. We allow for multiple candidate tract length distributions and select the best fitting distribution using the Bayesian Information Criterion (BIC). Using a mixture of two geometric components for the tract length distribution, we estimate that the smaller component has a mean of 16.9 bp (95% CI: [16.4, 17.0]), and the larger component has a mean of 724.7 bp (95% CI: [720.1, 728.7]). We further estimate the proportion of tracts from the second component to be 0.00525 (95% CI: [0.005, 0.00525]). After stratifying by crossover-hotspot overlap, we infer that tracts whose midpoints lie within crossover hotspots are, on average, longer than the remaining tracts.

## Author summary

Gene conversions are recombination events distinct from crossovers, in which alleles are transferred between homologous sequences within a short tract. Previous studies have investigated the lengths of gene conversion tracts using pedigree or sperm-typing data, but the number of gene conversion events that can be observed from these datasets is limited. In our study, we used almost one million detected gene conversion tracts from the ancestral history of UK Biobank participants to study the length distribution of these tracts. Within a gene conversion tract in the transmitting parent, alleles are only converted at heterozygous sites, so we cannot observe the full length of the gene conversion tract. To account for this in our method, we model the allele conversion probability separately for each detected tract. Our method allows for various distributions of the gene conversion tract length and is computationally efficient to handle all the tracts detected from the UK Biobank whole autosome data. Fitting a two-component model to shorter detected tracts that do not exceed 1.5 kb, we estimate the means of the two components to be 16.9 bp and 724.7 bp respectively. We further estimate the proportion of tracts from the second component to be 0.00525.

## Introduction

During meiosis, homologous chromosomes undergo genetic recombination resulting in the transfer of genetic material. Double strand breaks that occur during recombination are resolved in two distinct ways. Crossovers result in a long tract of DNA (typically spanning millions of base pairs) being exchanged between homologous chromosomes. On the other hand, non-crossover gene conversions typically result in a non-reciprocal transfer of alleles within a short tract [1]. These gene conversion events are thought to most commonly occur via the synthesis-dependent strand annealing mechanism, where a double stranded break is repaired by the invasion of a protruding 3’ end into the donor chromatid. Gene conversion events may also occur via the resolution of two Holliday junctions [2].

In this study, we use allele to refer to the nucleotide state at a single variant site. Gene conversions can be detected in humans by analyzing sequence data from pedigrees or sperm samples and identifying positions in which the allele of one homologous chromosome has been replaced by the other [1,3–5]. The length between these positions, where alleles are thought to have been converted by a gene conversion event, can be used to estimate the length of the gene conversion tract. We refer to the length between the outermost allele converted positions in the same gene conversion tract as the observed length of the gene conversion tract. Previous studies have focused on ascertaining these observed lengths to estimate the length of each gene conversion tract. Using SNP array and whole genome sequence data from 34 three-generation pedigrees, Williams et al. determined that tract lengths are in the order of 100–1,000 bp based on detected allele conversions [1]. Using three-generation pedigrees helps to distinguish between allele conversions and genotype errors. It can be difficult to distinguish between allele conversions and genotype errors when using two-generation pedigrees. Recently, HiFi PacBio long-read sequencing of sperm samples has enabled reliable observation of the furthest allele converted positions within a gene conversion tract [6–8].

Williams et al. further identified apparent clusters of gene conversion tracts spanning 20–30 kb, which may have resulted from discontinuous gene conversion events occurring in close proximity during the same meiosis [1]. This phenomenon has previously been referred to as complex gene conversions. Complex gene conversions as long as 100 kb were also found by Halldorsson et al. [5]. Complex gene conversions could arise from mechanisms such as GC-biased repair across long stretches of DNA [1]. In this study, we will focus on individual gene conversion tracts where the length spanning the outermost allele converted positions does not exceed 1.5 kb.

Efforts have been made to model the length distribution of gene conversion tracts using detected gene conversion tracts in humans and other species [9,10]. Recently, Palsson et al. detected 12,948 paternal and 15,712 maternal gene conversions transmitted to 5,420 trios in 2,132 Icelandic families [11]. Using their model, they estimated the mean length of gene conversion tracts to be 123 bp (95% CI: [94, 135]) and 102 bp (95% CI: [71, 125]) for paternal and maternal transmissions respectively [10,11].

Palsson et al. also found that the frequency of observed gene conversions was much higher in crossover recombination hotspots (22.4-fold and 13.7-fold for paternal and maternal transmissions respectively) [11]. Furthermore, gene conversion tracts associated with crossover events and complex gene conversion events have been found to be long (around 500 bp) relative to the mean tract length [8,12].

A large number of gene conversion tracts can be detected from biobank-scale sequence data using inferred identity-by-descent (IBD) clusters. Browning and Browning devised a method to use non-overlapping regions of each chromosome for detecting IBD clusters and gene conversions that have occurred on each IBD cluster [13]. Applying their method to whole autosome sequence data from 125,361 individuals from the UK Biobank, they found 9,313,066 allele conversions inferred to belong to 5,961,128 gene conversion tracts. To detect an allele conversion, this method requires at least two haplotypes within an IBD cluster to have the same alternate allele. This means that genotype errors will not be falsely identified as allele conversions, unless the same genotype error occurs twice in the same IBD cluster.

In our study, we propose a statistical method to model the length distribution of gene conversion tracts detected from the UK Biobank whole autosome data. In our method, we account for the difference in the true length of a gene conversion tract and its observed length, which we define as the length between the outermost allele converted positions in this gene conversion tract. The gene conversion tracts that we detect are from past transmissions in the population, for which the parental genotypes are not known. Allele conversions can only occur at heterozygous sites within a gene conversion tract in the transmitting parent, but we do not have access to the transmitting parent’s genotype data. This is not a problem in pedigree studies, where the positions of heterozygous sites in both parents are known. To appropriately account for the difference in the true and observed length of each gene conversion tract in our study without access to the transmitting parent’s genotype data, we assume that allele conversions occur with the same probability at each position within the same gene conversion tract. We estimate the allele conversion probability for each detected gene conversion tract using the heterozygosity rate of positions near the tract. Additionally, to account for the effects of linkage disequilibrium on the distribution of allele conversions, we found it necessary to exclude observed gene conversion tract lengths of one bp from our dataset, and we account for this exclusion in our analyses (see S1 Appendix).

We allow the length distribution of gene conversion tracts to follow a geometric random variable, a sum of two geometric random variables, or a mixture of two geometric components. A geometric distribution is appropriate if the gene conversion tract grows one bp at a time, and after each extension, there is a fixed probability that it continues extending to the next bp, independent of previous steps. This distribution has been found to accurately model the length distribution of gene conversion tracts in *Drosophila melanogaster* [14]. A sum of two geometric random variables is appropriate if the gene conversion tract extends outward in both directions from a central position, with each side following the same extension process as in the geometric case. Here, we assume that the probability of extending by one bp is the same in both directions. A mixture of two geometric components is a more flexible distribution that allows us to account for a wider tail in the tract length distribution compared to the previous two distributions. This mixture distribution has been used to model the length distribution of gene conversion tracts in previous studies. Using a mixture of two geometric components to model gene conversion tract lengths detected in a captive baboon colony, Wall et al. estimated the means of the two components to be 24 bp (95% CI: [18, 31]) and 4.3 kb (95% CI: [2.6, 4.9]) [9]. Furthermore, Palsson et al. similarly fit a mixture of two negative binomial distributions to shorter gene conversion tracts (less than 1 kb) detected in both human sexes [11]. For each tract length distribution, we derive a closed form expression for the distribution of observed tract lengths to efficiently calculate the joint likelihood for nearly one million detected gene conversion tracts during maximum likelihood estimation. After fitting our model assuming each tract length distribution, we use the Bayesian Information Criterion (BIC) to choose the best fitting tract length distribution [15].

We validate our method using two simulation studies. In the first simulation study, we fit our model on gene conversion tracts detected, using the IBD cluster method described in Browning and Browning (2024), from a coalescent simulation incorporating gene conversions with a mean tract length of 300 bp [13]. This coalescent simulation was conducted using *msprime* and includes crossover events and mutations [16]. Genotype errors were subsequently added to the simulated genomes. The details of this coalescent simulation and the subsequent detection of gene conversion tracts is described in Browning and Browning (2024) [13]. The purpose of applying our method to these detected gene conversion tracts is to assess our method’s ability to adjust for methodological constraints in the gene conversion detection method by Browning and Browning [13]. These constraints are described in more detail in the section, Detecting gene conversion tracts.

In our second simulation study, we draw gene conversion tract lengths from ten candidate distributions (five distributional families with two mean settings). One of these candidate distributions is a mixture distribution with parameter values that are nearly identical to what we inferred using detected gene conversion tracts from the UK Biobank whole autosome data. We overlay these gene conversion tracts on individual genomes obtained from the previous coalescent simulation to generate observed tract lengths. We then use our method to infer the mean length of gene conversion tracts using the generated observed tract lengths. The purpose of this simulation study is to assess the robustness of our method across a range of hypothetical gene conversion tract length distributions.

Finally, we apply our model to estimate the mean length of gene conversion tracts detected from the UK Biobank whole autosome data. In addition to estimating the mean length for all detected tracts, we stratify detected tracts based on whether they overlap with a crossover recombination hotspot and estimate the mean length separately for both sets of detected tracts.

## Materials and methods

### Ethics statement

The UK Biobank study was reviewed and approved by the North West Research Ethics Committee and all subjects gave informed consent [17].

### UK Biobank whole autosome data

We ran our analysis on whole autosome sequence data from 125,361 individuals from the UK Biobank, who identified themselves as ‘white British’ in the initial release of 150,119 sequenced genomes. The data were obtained under UK Biobank application number 19934, and the 150,119 genomes were phased using Beagle 5.4 [18,19].

### Detecting gene conversion tracts

We used gene conversion tracts previously detected in the UK Biobank whole autosome data using IBD clusters. IBD clusters are sets of haplotypes at a locus that have a recent common ancestor. If a recent gene conversion event transfers new alleles onto a lineage ancestral to a subset of these haplotypes, these new alleles will appear (at allele converted positions) in this subset of haplotypes in the IBD cluster. Browning and Browning use this signal to detect past gene conversion events [13]. Specifically, this method requires two haplotypes in the IBD cluster to have one allele, and another two haplotypes to have an alternative allele at the same position to detect an allele conversion. Using a coalescent simulation, Browning and Browning show that the false discovery rate of allele conversions using their method is low (around 1%) across different parameter settings. For full details of the IBD cluster and allele conversion detection method, as well as an overview of the simulation study used to calculate the false discovery rate of allele conversions, see Browning and Browning (2024) [13].

After allele conversions were detected, they were clustered to form detected gene conversion tracts. Allele conversions were considered to belong to the same gene conversion tract if they were located within 1.5 kb of each other, and if the membership of the two sub-clusters (representing the two alleles present in the IBD cluster) overlapped for the two allele conversions.

Across all the autosomes, 9,313,066 allele conversions were detected [13]. These allele conversions were inferred to belong to 5,961,128 detected gene conversion tracts. Furthermore, 4,943,183 (82.9%) of the detected gene conversion tracts were comprised of a single allele conversion [13]. 1,017,945 (17.1%) of the detected tracts were comprised of two or more allele conversions. We refer to the length spanning the outermost allele converted positions in a detected gene conversion tract as the observed tract length of the gene conversion tract. If a detected gene conversion tract is comprised of a single allele conversion, the observed tract length is one bp.

When estimating the mean length of gene conversion tracts from the observed tract lengths, we address two methodological constraints in the detection method, which are (i) allele conversions are only detected at variant positions with MAF greater than 5%, and (ii) observed gene conversion tract lengths cannot exceed 1.5 kb [13]. The former constraint was enforced to prevent mutations from creating the signal used to detect allele conversions. We adjust for this by calibrating allele conversion probabilities in our method (see the section, Estimating the allele conversion probability for each detected tract). The latter constraint exists because of how the gene conversion detection method uses overlapping windows. Observed tracts that are longer than 1.5 kb may not be fully contained in any detection window (regions where allele conversions are detected, as opposed to IBD clusters) and thus may be missed.

We label the observed tract lengths of all detected gene conversion tracts as $\left\{l_{j}|j=\text{1},\ldots ,m\right\}$. To account for the constraint on observed tract lengths, we exclude any observed tract lengths exceeding 1.5 kb when estimating the mean gene conversion tract length. This results in the exclusion of 141,361 tracts (2.4% of all detected tracts). We also exclude observed tract lengths of one bp prior to estimation, because our model assigns a higher probability mass at one bp compared to what we observe in the data (see S1 Appendix). This is likely because we do not account for linkage disequilibrium in our model. Although we exclude observed tract lengths of one bp when estimating the mean gene conversion tract length, we use the proportion of one bp observed tracts to understand the effect of linkage disequilibrium on the distribution of observed tract lengths (see S1 Appendix). We appropriately account for the omission of these tracts in our model by truncating the marginal distribution of observed tract lengths (derived in a later section) at one bp and 1.5 kb. After removing both detected tracts of 1 bp and those exceeding 1.5 kb, we are left with 876,584 detected tracts. Although excluding these tracts reduces the amount of data used in the estimation procedure, results from our simulation studies suggest that the resulting estimator from the truncated model has small bias under correct specification of the gene conversion tract length distribution.

### Definitions and overview of model

We model N, the length of a gene conversion tract, as a geometric random variable, a sum of two independent and identically distributed geometric random variables, or a mixture of two geometric components. We use ϕ to represent the mean of N. We further let L be a random variable representing the observed tract length of a gene conversion tract, which is the length spanning the outermost allele converted positions within the gene conversion tract. The event L=0 represents no allele conversions occurring within the tract, and L=1 represents one allele conversion occurring within the tract. In the following sections, we derive the conditional distribution of L given N and the marginal distribution of L. We further describe the procedure we use to obtain a maximum likelihood estimate of ϕ, $\hat{\phi }$, using the observed tract lengths $\left\{l_{j}|j=\text{1},\ldots ,m\right\}$ detected from the UK Biobank whole autosome data.

### The distribution of the observed tract length conditional on the gene conversion tract length

The observed tract length of a gene conversion tract, represented by the random variable L, depends on where allele conversions occur on the gene conversion tract. We will first assume that allele conversions happen with probability ψ at every position within some gene conversion tract that is exactly n bp long. Under this scenario, the following conditional distribution has previously been derived [20].

$P\left(L=l|N=n\right)=\left\{ \begin{array}{c} (\text{1}-\psi {)}^{n}\text{if}\;l=\;\text{0}\\ n\psi (\text{1}-\psi {)}^{n-\text{1}}\text{if}l=\text{\textasciitilde{}1}\\ (n-l+\text{1})\psi^{\text{2}}(\text{1}-\psi {)}^{n-l}\text{if}\text{2}\leq l\leq n. \end{array}\right.$

In the probability above, we conditioned on the gene conversion tract length, represented by the random variable N, being n bp long. Obtaining an observed tract length of zero bp is equivalent to allele conversions not occurring within the gene conversion tract, which happens with a probability of $(\text{1}-\psi {)}^{n}$. Next, obtaining an observed tract length of one bp is equivalent to an allele conversion occurring at exactly one position within the gene conversion tract. There are n possible positions in which the allele conversion can occur, and each configuration happens with a probability of ${\psi (\text{1}-\psi )}^{n-\text{1}}$. Lastly, to obtain an observed tract length of l bp, where 2≤𝓁≤n, we need to observe two allele conversions that span exactly l positions, and allele conversions cannot occur at the n−l positions between the two tract endpoints and the nearest allele conversion. There are n−l+1 ways to overlay these two allele conversions on the gene conversion tract, and each configuration occurs with a probability of ${\psi^{\text{2}}(\text{1}-\psi )}^{n-l}$.

### Deriving the marginal distribution of the observed tract length

If the gene conversion tract length N is drawn from geometric distribution with mean ϕ, we have,


$$P(N=n)={\left(\text{1}-\frac{\text{1}}{\phi }\right)}^{n-\text{1}}\frac{\text{1}}{\phi }.$$

Letting λ=1/ϕ,


$$P\left(L=l\right)=\sum_{n=l}^{\infty }P\left(L=l|N=n\right)P(N=n)$$

$$=\left\{ \begin{array}{c} @l\frac{\lambda (\text{1}-\psi )}{\lambda +\psi -\lambda \psi }\text{if}l=\text{0}\\ \frac{\lambda \psi }{(\lambda +\psi -\lambda \psi {)}^{\text{2}}}\text{if}l=\text{1}\\ \frac{\lambda (\text{1}-\lambda {)}^{l-\text{1}}\psi^{\text{2}}}{(\lambda +\psi -\lambda \psi {)}^{\text{2}}}\text{if}l\geq \text{2}. \end{array}\right.$$

This is the marginal distribution of the observed tract length L. A closed form expression for L was not derived previously, but this form is crucial for accelerating likelihood computations, given that we compute the joint likelihood of nearly one million observed tract lengths during maximum likelihood estimation. We further truncate this distribution to appropriately model observed tract lengths detected in the UK Biobank sequence data using the multi-individual IBD method [13]. Recall that we only retain observed tract lengths between 2 and 1,500 bp during estimation, so we account for this by truncating the distribution of L between 2 and 1,500 bp.

We have


$$P(\text{2}\leq L\leq \text{1}\text{5}\text{0}\text{0})=\sum_{l=\text{2}}^{\text{1}\text{5}\text{0}\text{0}}\frac{\lambda (\text{1}-\lambda {)}^{l-\text{1}}\psi^{\text{2}}}{(\lambda +\psi -\lambda \psi {)}^{\text{2}}}=\frac{\psi^{\text{2}}[(\text{1}-\lambda )-(\text{1}-\lambda {)}^{\text{1}\text{5}\text{0}\text{0}}]}{(\lambda +\psi -\lambda \psi {)}^{\text{2}}}.$$

Then,


$$P\left(L=l|\text{2}\leq L\leq \text{1}\text{5}\text{0}\text{0}\right)=\frac{P\left(L=l\right)}{P(\text{2}\leq L\leq \text{1}\text{5}\text{0}\text{0})}=\frac{\lambda (\text{1}-\lambda {)}^{l-\text{1}}}{\left[(\text{1}-\lambda )-(\text{1}-\lambda {)}^{\text{1}\text{5}\text{0}\text{0}}\right]}.$$

Notice that conditioning on 2≤L≤1500 removed the parameter ψ from our model.

As mentioned earlier, $\left\{l_{j}|j=\text{1},\ldots ,m\right\}$ represents the observed tract lengths in our dataset. When fitting the model, we use the filtered set of observed tract lengths, $\left\{l_{j}|j=\text{1},\ldots ,m,\text{2}\leq l_{j}\leq \text{1}\text{5}\text{0}\text{0}\right\}$. Henceforth, we will also index our random variable L using j. $L_{j}$ represents the random variable corresponding to the observed tract length of detected gene conversion tract j in our dataset. We have,


$$P\left(L_{j}=l_{j}|\text{2}\leq L_{j}\leq \text{1}\text{5}\text{0}\text{0},\lambda \right)=\frac{\lambda (\text{1}-\lambda {)}^{l_{j}-\text{1}}}{\left[(\text{1}-\lambda )-(\text{1}-\lambda {)}^{\text{1}\text{5}\text{0}\text{0}}\right]}.$$

We also consider two alternative distributions for N: a sum of two independent and identically distributed geometric random variables, and a mixture of two geometric components. We derive $P\left(L_{j}=l_{j}|\text{2}\leq L_{j}\leq \text{1}\text{5}\text{0}\text{0}\right)$ under both settings (see S2 Appendix). Under these settings, $P\left(L_{j}=l_{j}|\text{2}\leq L_{j}\leq \text{1}\text{5}\text{0}\text{0}\right)$ depends on $\psi_{j}$, so we estimate $\psi_{j}$ for each tract j before estimating ϕ. The procedure to estimate $\psi_{j}$ for each tract j is described in the following section.

### Estimating the allele conversion probability for each detected tract

Recall that $\psi_{j}$ represents the probability that an allele conversion will occur at each position within detected gene conversion tract j. When N is a sum of two geometric random variables or a mixture of two geometric components, the likelihood of the observed tract length for detected gene conversion tract j, $P\left(L_{j}=l_{j}|\text{2}\leq L_{j}\leq \text{1}\text{5}\text{0}\text{0}\right)$, depends on $\psi_{j}$ (see S2 Appendix), so we need to estimate $\psi_{j}$ for j=1,…,m to obtain a maximum likelihood estimate for the mean gene conversion tract length ϕ.

Allele conversions occur at positions within each gene conversion tract where the individual is heterozygous. Therefore, the probability that a randomly selected individual from the population is heterozygous at a given position can be used to estimate the probability that an allele conversion will happen at this position, once it is included in a gene conversion tract. However, it is difficult to derive a closed form expression for the marginal distribution of L when we only allow allele conversions to occur at SNV positions, and with differing rates at each SNV position. Thus, we let allele conversions occur with the same probability $\psi_{j}$ at all positions within detected gene conversion tract j. We use the average heterozygosity rate of positions near detected tract j to estimate $\psi_{j}$.

Letting $a_{j}$ and $b_{j}$ ($a_{j}\leq b_{j}$) represent the positions on the chromosome corresponding to the outermost allele converted positions within detected gene conversion tract j, we average the heterozygosity rate across the set of positions $\left[a_{j}-\text{5}\text{0}\text{0}\text{0},b_{j}+\text{5}\text{0}\text{0}\text{0}\right]$ to estimate $\psi_{j}$:


$${\hat{\psi }}_{j}=\frac{\text{1}}{b_{j}-a_{j}+\text{1}\text{0}\text{0}\text{0}\text{1}}\sum_{i=a_{j}-\text{5}\text{0}\text{0}\text{0}}^{b_{j}+\text{5}\text{0}\text{0}\text{0}}\text{2}p_{i}(\text{1}-p_{i}).$$

Here, $p_{i}$ denotes the MAF of position i on the chromosome in which the gene conversion event occurred. $p_{i}$ is calculated using the sample of 125,361 White British individuals from the UK Biobank. Variants with MAF less than 5% were excluded when detecting allele conversions, so we cannot observe allele conversions at these positions (see the section, Detecting gene conversion tracts). Therefore, if the MAF is less than 5% at position i, we set $p_{i}=\text{0}$. The formula 2p(1−p) for heterozygosity at a position assumes that Hardy-Weinberg equilibrium holds, which is a reasonable approximation for common variants in a relatively homogeneous population.

If either $a_{j}-\text{5}\text{0}\text{0}\text{0}$ or $b_{j}+\text{5}\text{0}\text{0}\text{0}$ exceeds the end of the chromosome, the averaging only takes place within the bounds of the chromosome (e.g., if $a_{j}=\text{1}\text{0}\text{0}$ and $b_{j}=\text{2}\text{0}\text{0}$, we only average the heterozygosity rate from positions 1–5,200).

### Maximum likelihood estimation of the mean gene conversion tract length

Given observed tract lengths $\left\{l_{j}|j=\text{1},\ldots ,m\right\}$, we propose the following maximum likelihood estimator for ϕ, the mean gene conversion tract length, when the gene conversion tract length N is drawn from a geometric distribution. Recall that the version of the model in which N is geometric was parameterized by λ=1/ϕ, but we can simply maximize with respect to ϕ. In other words,


$$\hat{\phi }=\underset{\phi }{\text{argmax}}\sum_{j\in I_{\text{2}}^{\text{1}\text{5}\text{0}\text{0}}}\text{log}P(L_{j}=l_{j}|\text{2}\leq L_{j}\leq \text{1}\text{5}\text{0}\text{0},\phi ),$$

where $I_{\text{2}}^{\text{1}\text{5}\text{0}\text{0}}=\left\{j=\text{1},\ldots ,m|\text{2}\leq l_{j}\leq \text{1}\text{5}\text{0}\text{0}\right\}$. When N is a sum of two geometric random variables, we parameterize the distribution of L using γ=2/ϕ (see S2 Appendix). Unlike the geometric case, our marginal distribution of $L_{j}$ truncated between 2 and 1,500 still depends on $\psi_{j}$, so for each j, we plug in our estimated ${\hat{\psi }}_{j}$ in place of $\psi_{j}$. Then, we can again maximize with respect to ϕ:


$$\hat{\phi }=\underset{\phi }{\text{argmax}}\sum_{j\in I_{\text{2}}^{\text{1}\text{5}\text{0}\text{0}}}\text{log}P(L_{j}=l_{j}|\text{2}\leq L_{j}\leq \text{1}\text{5}\text{0}\text{0},\phi ,\psi_{j}={\hat{\psi }}_{j}).$$

When N is a mixture of two geometric components, we have three unknown parameters $\phi_{\text{1}}$, $\phi_{\text{2}}$, and $w_{\text{1}}$, which represent the mean of the first component, the mean of the second component, and the mixing proportion of the first component (see S2 Appendix). Again, our marginal distribution of $L_{j}$ truncated between 2 and 1,500 still depends on $\psi_{j}$, so for each j, we plug in our estimated ${\hat{\psi }}_{j}$ in place of $\psi_{j}$. Then, we can maximize with respect to $\phi_{\text{1}}$, $\phi_{\text{2}}$, and $w_{\text{1}}$:


$${\hat{\phi }}_{\text{1}},{\hat{\phi }}_{\text{2}},{\hat{w}}_{\text{1}}=\underset{\phi_{\text{1}},\phi_{\text{2}},w_{\text{1}}}{\text{argmax}}\sum_{j\in I_{\text{2}}^{\text{1}\text{5}\text{0}\text{0}}}\text{log}P(L_{j}=l_{j}|\text{2}\leq L_{j}\leq \text{1}\text{5}\text{0}\text{0},\phi_{\text{1}},\phi_{\text{2}},w_{\text{1}},\psi_{j}={\hat{\psi }}_{j}).$$

To find the argmax when N is geometric or a sum of two geometric random variables, we use the L-BFGS-B algorithm implemented in the scipy.optimize.minimize function from the SciPy Python library [21]. When N is a mixture of two geometric components, we define a grid for $w_{\text{1}}$ ranging from 0.002 to 0.5, using increments of 0.00025 between 0.002 and 0.01, and increments of 0.05 between 0.05 and 0.5. We chose a finer grid at smaller values of $w_{\text{1}}$ because preliminary analyses of observed tract lengths from the UK Biobank whole autosome data consistently inferred $w_{\text{1}}$ to be close to zero. Then, for each $w_{\text{1}}$ value in the grid, we again ran the L-BFGS-B algorithm from four starting values of $\left(\phi_{\text{1}},\phi_{\text{2}}\right):(\text{0}.\text{0}\text{0}\text{0}\text{5},\text{0}.\text{0}\text{0}\text{0}\text{5}),(\text{0}.\text{0}\text{0}\text{0}\text{5},\text{0}.\text{1}),(\text{0}.\text{1},\text{0}.\text{0}\text{0}\text{0}\text{5}),$ and (0.1,0.1). Multiple starting values were used because the likelihood of $\left(\phi_{\text{1}},\phi_{\text{2}}\right)$ (fixing $w_{\text{1}}$) appeared to have multiple local maxima. The final maximum likelihood estimates were selected as the set of $\left(w_{\text{1}},\phi_{\text{1}},\phi_{\text{2}}\right)$ values achieving the highest joint likelihood across all grid points of $w_{\text{1}}$ and starting values of L-BFGS-B.

To choose between the three distributions of N, we propose calculating the Bayesian Information Criterion (BIC) under each version of the model. Lower BIC indicates that the distribution of N that is used is a better fit to the data.

### Bootstrap confidence intervals

We calculate 95% bootstrap confidence intervals for ϕ ($w_{\text{1}},\phi_{\text{1}},\phi_{\text{2}}$ in the case where N is a mixture of two geometric components). We denote the number of detected gene conversion tracts with observed tract length between 2 and 1,500 bp as $\left|I_{\text{2}}^{\text{1}\text{5}\text{0}\text{0}}\right|$. To obtain each bootstrap sample, we sample with replacement $\left|I_{\text{2}}^{\text{1}\text{5}\text{0}\text{0}}\right|$ observed tract lengths from the set $\left\{l_{j}|j=\text{1},\ldots ,m,\text{2}\leq l_{j}\leq \text{1}\text{5}\text{0}\text{0}\right\}$. Each bootstrap sample consists of the set of observed tract lengths $\left\{l_{j}\right\}$ and allele conversion probabilities $\left\{\psi_{j}\right\}$ corresponding to the resampled indices.

We refit our model to 500 bootstrap samples and obtain a new maximum likelihood estimate of ϕ (or $w_{\text{1}},\phi_{\text{1}},\phi_{\text{2}}$ in the case where N is a mixture of two geometric components) for each bootstrap sample. We take the 0.025 and 0.975 quantiles of the resulting bootstrap distributions and use this as the bounds of our 95% bootstrap confidence intervals.

### Simulation study 1

We use simulated data described in Browning and Browning (2024) [13]. 20 regions of length 10 Mb were generated for 125,000 individuals using the coalescent simulator *msprime* v1.2 [16]. The demographic model for the simulation was an exponentially growing population with an initial size of 10,000 and a growth rate of 3% per generation for the past 200 generations. To simulate recombination and mutation, a crossover rate of 1 cM/Mb and a mutation rate of $\text{1}.\text{5}\times {\text{1}\text{0}}^{-\text{8}}$ per bp per meiosis were used. The mutation rate used is similar to previously inferred mutation rates using IBD segments [22,23]. Gene conversions were simulated with an initiation rate of 0.02 per Mb and gene conversion lengths were simulated from a geometric distribution with a mean tract length of 300 bp. Uncalled deletions and genotype errors were added to the resulting genomes [13]. The details of this coalescent simulation, the procedure used to add uncalled deletions and genotype errors, and the subsequent detection of gene conversion tracts is described in Browning and Browning (2024) [13]. The multi-individual IBD analysis detected 284,838 allele conversions belonging to 226,007 detected gene conversion tracts across the 20 regions. We fit our model to the detected gene conversion tracts in each of the 20 regions to estimate the mean gene conversion tract length in each region. For the purposes of this simulation study, we refer to the detected gene conversion tracts in each region as a separate replicate dataset. We refer to fitting our model to the detected gene conversion tracts in each of the 20 regions as a separate replicate of this simulation study.

We fit our model under all three distributions for the true tract length (geometric, sum of two geometric random variables, and mixture of two geometric components). Because the true tract lengths in this simulation study are drawn from a geometric distribution, we are interested in whether the version of the model in which the tract length is geometric will be favored using BIC.

*msprime* only allows gene conversion tract lengths to be drawn from a geometric distribution [16]. Thus, to test the robustness of our method to different tract length distributions, we run an additional simulation study drawing gene conversion tract lengths from various distributions, including a mixture of two geometric components.

### Simulation study 2

We conduct an additional simulation study to evaluate how well our method recovers the mean tract length ϕ when gene conversion tract lengths are drawn from candidate distributions. We simulate observed tract lengths $\left\{l_{j}|j=\text{1},\ldots ,m\right\}$ using ten distributions (five distributional families and two mean settings) for the length distribution of gene conversion tracts, shown in Table 1. We plot these distributions in S3 Fig. The mixture distribution with a mean tract length of 21 bp has parameter values nearly identical to what we estimated using detected gene conversion tracts from the UK Biobank whole autosome data (see Results).

**Table 1:** Summary of distributions and parameter values used to simulate gene conversion tract lengths.

Distribution	Parameters	True mean (bp)
Geometric	ϕ=21	21
Geometric	ϕ=100	100
Sum of two geometric	Each ϕ=21/2	21
Sum of two geometric	Each ϕ=100/2	100
Sum of three geometric	Each ϕ=21/3	21
Sum of three geometric	Each ϕ=100/3	100
Discrete uniform	Support: 1,2,3,…,41	21
Discrete uniform	Support: 1,2,3,…,199	100
Mixture of geometric	$\phi_{\text{1}}=\text{7}\text{2}\text{5},\phi_{\text{2}}=\text{1}\text{7}.\text{5},w_{\text{1}}=\text{0}.\text{0}\text{0}\text{5}$	21
Mixture of geometric	$\phi_{\text{1}}=\text{7}\text{0}\text{0},\phi_{\text{2}}=\text{6}\text{8}.\text{4},w_{\text{1}}=\text{0}.\text{0}\text{5}$	100

Recall that in the previous coalescent simulation, we generated 20 regions of length 10 Mb for 125,000 individuals. In this simulation study, we generate observed tract lengths by simulating gene conversion tracts on the first region (of the 20 regions) from the previous coalescent simulation.

We first sample 10,000 individuals with replacement from the 125,000 individuals. We simulate 100 gene conversion tracts on each resampled individual using the following steps:

1. We randomly select a starting position for each of the 100 gene conversion tracts, chosen uniformly across the 10 Mb region.
2. We draw the length of each gene conversion tract from one of the ten distributions in Table 1.
3. We determine the observed tract length for each gene conversion tract as the length spanning the outermost heterozygous markers within the simulated gene conversion tract.

This procedure results in 1,000,000 observed tract lengths, most of which are unobservable due to the absence of heterozygous markers within the corresponding gene conversion tracts. For each of the ten distributions listed in Table 1, we repeat this procedure (sampling individuals and sampling tracts on each individual) to obtain 100 sets of 1,000,000 observed tract lengths. Then, we apply our method on each set to estimate the mean tract length ϕ. We use all three distributions of the true tract length N to fit our model (geometric, sum of two geometric random variables, and a mixture of two geometric components).

The number of observed tract lengths between 2 and 1,500 bp differs for each set. Furthermore, when the mean of the tract length distribution is 21 bp, we observe fewer tract lengths between 2 and 1,500 bp than when the mean is 100 bp. This is because shorter tracts are less likely to overlap at least two heterozygous markers. To make sure that we are using the same sample size for estimation, we sample 1,000 observed tract lengths between 2 and 1,500 bp in each of our 100 sets for distributions where the true mean is 100 bp in Table 1. Likewise, we sample 50 observed tract lengths between 2 and 1,500 bp in each set for distributions where the true mean is 21 bp.

For each set of observed tract lengths, and for each assumed distribution for the true tract length N, we obtain both a point estimate and a 95% bootstrap confidence interval for the mean tract length. We again calculate BIC for each assumed distribution for N to evaluate the performance of the BIC-selected model.

### UK Biobank analysis

We previously described how we obtain the observed tract lengths of all detected gene conversion tracts from the UK Biobank whole autosome data, denoted $\left\{l_{j}|j=\text{1},\ldots ,m\right\}$. We fit our model on this dataset, using all three tract length distributions (geometric, sum of two geometric random variables, and mixture of two geometric components). We further compare model fit under each of these distributions using BIC.

In addition, we run a stratified analysis, stratifying observed tract lengths based on whether they overlapped with a crossover hotspot. To avoid ascertainment bias, where longer tracts are more likely to overlap a crossover hotspot by chance, we defined overlap based on whether the midpoint of the detected gene conversion tract was inside a crossover hotspot. To define crossover hotspots, we use the deCODE genetic map from Halldorsson et al. and follow their definition of crossover hotspots as regions with crossover rates exceeding ten times the genome-wide average [24].

We calculate local crossover rates between nearby SNV positions on each chromosome by dividing the genetic distance between the two SNV positions by their physical distance. Initially, we calculate the local crossover rate between the first SNV position in the genetic map, and the SNV position closest to it that is distant by at least 2 kb. We next calculate the local crossover rate between this newly identified SNV position and the SNV position closest to it that is distant by at least 2 kb. We repeat this process until the last SNV position on this chromosome is included in a local crossover rate calculation, or until we cannot identify further SNV positions that are at least 2 kb away.

If the local crossover rate between two SNV positions is more than ten times the genome-wide average, we classify the region spanning these SNV positions as a crossover hotspot. We stratify the observed tract lengths $\left\{l_{j}|j=\text{1},\ldots ,m\right\}$ based on whether the midpoint of the corresponding detected gene conversion tract was inside a crossover hotspot. We then fit our model, separately for each set of tracts. We again use all three tract length distributions to fit the model in this stratified analysis, and compare model fit using BIC.

## Results

### Simulation study 1

We fit our model to the observed tract lengths from each replicate of the simulation study. The number of observed tract lengths between 2 bp and 1.5 kb across the 20 replicates ranged from 2,005–2,314. Recall that a geometric distribution with mean 300 bp was used to simulate gene conversion tract lengths in this simulation study. We estimate the mean tract length under all three tract length distributions (geometric, sum of two geometric random variables, and mixture of two geometric components).

**Fig 1:**
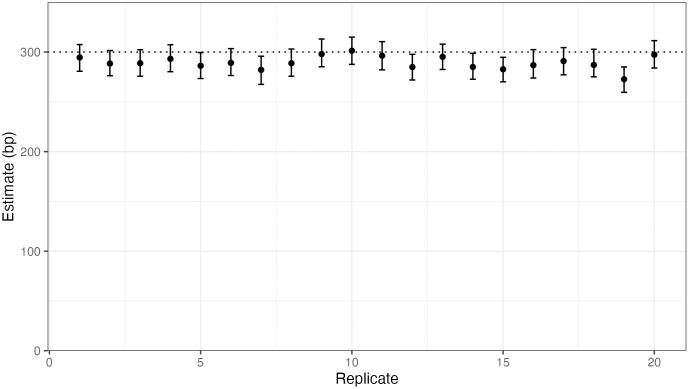
The estimated mean gene conversion tract length under the geometric setting across replicate simulations. The dotted horizontal line represents the true mean gene conversion tract length. Gene conversion tract lengths were simulated using a geometric distribution. We plot our estimate and 95% bootstrap confidence interval under the geometric setting for each replicate simulation.

Estimates and confidence intervals using the geometric setting are shown in Fig 1. The average estimate of the mean tract length across the 20 replicates is 289.5 bp under the geometric setting, which is slightly lower than the true mean of 300 bp used to simulate the gene conversion tracts. Under the geometric setting, the true mean of 300 bp is contained in our 95% bootstrap confidence intervals in 14 out of the 20 replicates.

**Fig 2:**
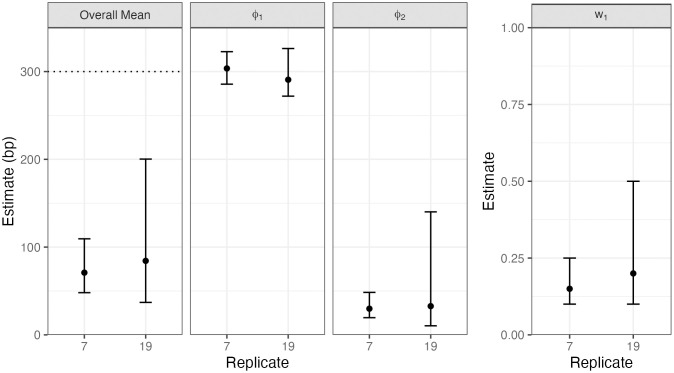
Parameter estimates for two replicates using the mixture distribution. The dotted horizontal line represents the true mean gene conversion tract length. We plot the estimated parameter values with 95% bootstrap confidence intervals for each replicate simulation.

The geometric setting results in the smallest BIC in 18 out of the 20 replicates. For the remaining two replicates, BIC is lowest when gene conversion tract lengths are assumed to be drawn from a mixture of two geometric components. Estimates for these two replicates using the mixture setting are shown in Fig 2.

For these two replicates, we see that the mixture setting underestimates the overall mean of 300 bp. Notice that the mean of the first component is estimated to be close to 300 bp for these replicates, but the mean of the second component is estimated to be much lower. The mixing proportion of the first component is estimated to be between 0.15 and 0.2 across the two replicates. 95% confidence intervals for parameters tend to be wide, except for the mean of the first component. For these two replicates, we also plot the model fit for the geometric and mixture models along with the empirical distribution of observed tract lengths (S4 Fig). Although the mixture model with the estimated parameters has a slightly longer tail than the geometric model, the number of observed tract lengths is too small to fully characterize the tail of the empirical distribution. The difference in BIC between the mixture model and the geometric model (which had the next lowest BIC) was 11.6 and 3.2 for replicate 7 and 19 respectively.

Across all 20 replicates, the difference in BIC between the geometric and mixture settings (positive values preferring the mixture setting) range from 11.6 to -15.5.

### Simulation study 2

In Table 2, we report the empirical bias and standard deviation of our estimate of the mean, as well as the empirical coverage probability of our 95% confidence interval of the mean under all assumed distributions of the true tract length across 100 sets of observed tract lengths generated using each of the ten distributions in Table 1. Recall that we kept the sample size constant at 50 and 1,000 observed tracts (with lengths between 2 and 1,500 bp) for distributions with mean 21 bp and 100 bp respectively. Under the BIC-selected distributional setting, we use the estimate and confidence interval from the distributional setting with the smallest Bayesian Information Criterion (BIC) value in each of the 100 replicates.

**Table 2:** Results from simulation study to assess robustness. We assess the performance of our method under each distribution that we use to simulate the true tract lengths (first column) and the chosen setting of the tract length distribution (second column). We report the empirical bias (third column) and standard deviation (fourth column) of our estimate of the mean, as well as the empirical coverage of our 95% confidence interval (fifth column) across 100 replicates of the simulation study. Under the BIC-selected setting, we use the estimate and confidence interval from the distributional setting with the smallest Bayesian Information Criterion (BIC) value in each of the 100 replicates.

Distribution	Assumed Distribution	Bias	SD	Coverage
Geom (mean: 21 bp)	Geom	0.0	3.1	0.92
	Geom2	10.7	4.6	0.20
	Mixture	-3.9	5.2	0.82
	BIC-selected	5.5	6.9	0.50
Geom (mean: 100 bp)	Geom	-2.0	3.3	0.87
	Geom2	46.6	4.9	0.00
	Mixture	-14.1	25.8	0.79
	BIC-selected	8.4	19.1	0.71
Geom2 (mean: 21 bp)	Geom	-6.7	1.9	0.08
	Geom2	0.4	2.8	0.92
	Mixture	-7.5	2.6	0.07
	BIC-selected	-1.2	4.2	0.71
Geom2 (mean: 100 bp)	Geom	-33.5	2.1	0.00
	Geom2	-0.7	3.2	0.94
	Mixture	-33.5	2.1	0.00
	BIC-selected	-1.6	6.6	0.91
Geom3 (mean: 21 bp)	Geom	-9.3	1.4	0.00
	Geom2	-3.5	2.1	0.53
	Mixture	-9.6	1.5	0.00
	BIC-selected	-4.2	2.7	0.46
Geom3 (mean: 100 bp)	Geom	-43.9	1.6	0.00
	Geom2	-16.5	2.4	0.00
	Mixture	-43.9	1.6	0.00
	BIC-selected	-16.5	2.4	0.00
Uniform (mean: 21 bp)	Geom	-10.3	1.1	0.00
	Geom2	-4.9	1.7	0.17
	Mixture	-10.3	1.1	0.00
	BIC-selected	-5.0	1.8	0.17
Uniform (mean: 100 bp)	Geom	-48.3	1.2	0.00
	Geom2	-23.5	1.7	0.00
	Mixture	-48.3	1.2	0.00
	BIC-selected	-23.5	1.7	0.00
Mixture (mean: 21 bp)	Geom	382.7	95.4	0.00
	Geom2	566.3	121.1	0.00
	Mixture	7.7	37.8	0.96
	BIC-selected	20.2	91.0	0.92
Mixture (mean: 100 bp)	Geom	256.0	15.6	0.00
	Geom2	422.5	20.8	0.00
	Mixture	-22.4	29.2	1.00
	BIC-selected	-15.5	47.1	0.98

In Table 3, we summarize the number of times each distribution of N was preferred by BIC across the 100 sets of observed tract lengths generated under each distribution in Table 1.

**Table 3:** Number of replicates each distributional setting was selected by the Bayesian Information Criterion (BIC). For each of the five data-generating distributions, we simulated 100 sets of observed tract lengths. We then counted how many times each tract length distribution was selected as the best fitting distribution based on BIC.

Distribution	Assumed Distribution	Times Selected by BIC
Geom (mean: 21 bp)	Geom	48
	Geom2	52
	Mixture	0
Geom (mean: 100 bp)	Geom	78
	Geom2	22
	Mixture	0
Geom2 (mean: 21 bp)	Geom	23
	Geom2	77
	Mixture	0
Geom2 (mean: 100 bp)	Geom	3
	Geom2	97
	Mixture	0
Geom3 (mean: 21 bp)	Geom	12
	Geom2	88
	Mixture	0
Geom3 (mean: 100 bp)	Geom	0
	Geom2	100
	Mixture	0
Uniform (mean: 21 bp)	Geom	1
	Geom2	99
	Mixture	0
Uniform (mean: 100 bp)	Geom	0
	Geom2	100
	Mixture	0
Mixture (mean: 21 bp)	Geom	4
	Geom2	0
	Mixture	96
Mixture (mean: 100 bp)	Geom	2
	Geom2	0
	Mixture	98

### UK Biobank analysis

We applied our estimation method to the observed tract lengths detected from the UK Biobank whole autosome data. The BIC is lowest (indicating best fit) under the setting where the true tract length distribution is assumed to be a mixture of two geometric components (11,860,358). The BIC for the geometric and sum of two geometric settings were 12,201,928 and 12,268,165 respectively. The difference in BIC between the mixture setting and the geometric setting, which had the next lowest BIC, was 341,569, providing strong evidence in favor of the mixture setting.

**Fig 3:**
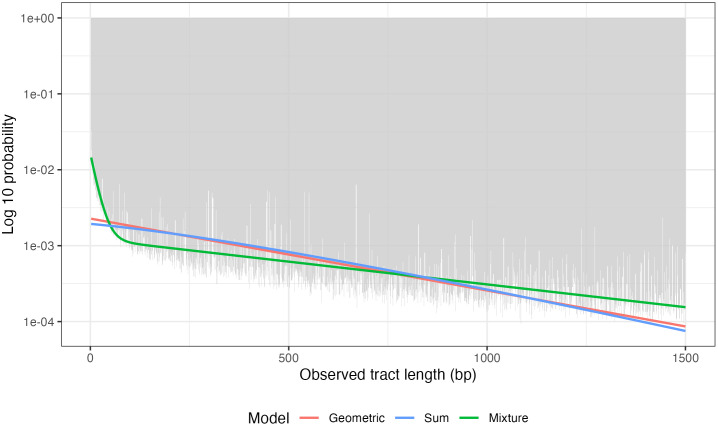
Empirical distribution of observed tract lengths and model fits. We plot the inferred distribution of observed tract lengths under our three tract length distributions, and the empirical distribution of observed tract lengths in our dataset. Probabilities are in the log 10 scale. To obtain the model fits, we average the probability mass at l=1,2,…,1500 across estimated allele conversion probabilities for each tract (${\hat{\psi }}_{j}$).

When assuming that gene conversion tract lengths are a mixture of two geometric components, we estimate the mixing proportion for the first component to be 0.00525 (95% CI: [0.005, 0.00525]). We estimate the mean of the first and second components to be 724.7 bp (95% CI: [720.1, 728.7]) and 16.9 bp (95% CI: [16.4, 17.0]) respectively. We estimate the overall mean to be 20.6 bp (95% CI: [19.9, 20.7]). In Fig 3, we show the inferred distribution of observed tract lengths along with the empirical distribution of observed tract lengths. Because our distribution of L depends on the allele conversion probability, which differs for each observed tract, we take the average probability mass across the set of allele conversion probabilities in our dataset at each value of l=1,2,…,1500 to plot the inferred distribution. We see that the mixture model better fits the observed tract lengths in our dataset, especially at lower and higher values of the observed tract length.

**Fig 4:**
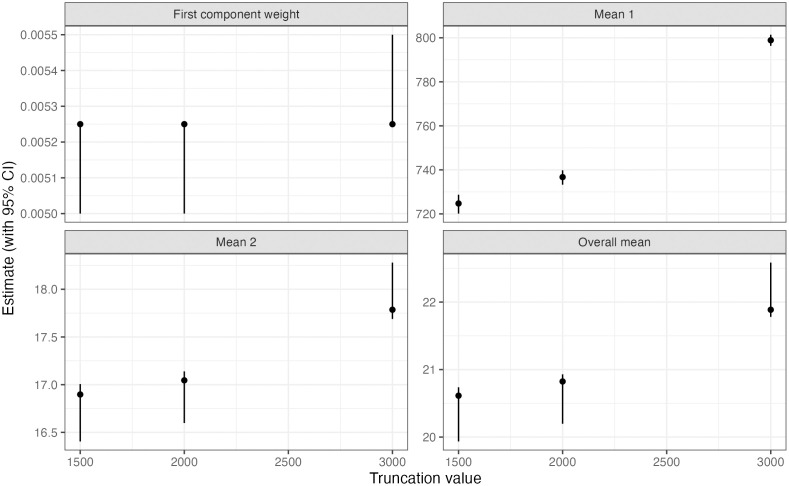
Parameter estimates under the mixture model across different truncation settings. For truncation boundaries of 1.5 kb, 2 kb, and 3 kb, we refit the truncated mixture model (excluding observed tracts exceeding the bound) and plot estimates with 95% confidence intervals for the mean length of component 1 ($\phi_{\text{1}}$), component 2 ($\phi_{\text{2}}$), the mixing proportion of component 1 ($w_{\text{1}}$), and the overall mean.

We further reran the above analysis changing the truncation boundary to 2 kb and 3 kb from the original 1.5 kb. Recall that we fit a truncated model, excluding all observed tracts that exceed the truncation bound (see Methods). We saw that our estimate of the overall mean was less sensitive to changing the truncation bound for the mixture model, compared to when we assumed the distribution of true tract lengths to follow a geometric distribution or sum of two geometric random variables. For the mixture model, our estimate of the overall mean ranged from 20.6 bp to 21.9 bp across the three truncation boundaries. When we assumed the distribution to follow a geometric or sum of two geometric random variables, our estimate of the mean ranged from 459 bp to 607 bp and 649 bp to 873 bp respectively. We further found that if we do not truncate the mixture model above, the probability mass above 1.5 kb is 0.10, which is similar to the proportion of observed tract lengths that exceed 1.5 kb (0.14). In Fig 4, we plot our point estimates and 95% confidence intervals for the mean of each component and the mixing proportion of the first component under the mixture model across the three truncation settings.

For the stratified analysis, we calculated the genome-wide average crossover rate to be 1.23 cM/Mb. We classify any regions exceeding ten times this rate as a crossover hotspot. Of the 876,584 tracts detected from the UK Biobank sequence data, the midpoints of 290,766 (33.2%) were contained within a crossover hotspot. For both tract sets, the set of tracts with midpoint in a crossover hotspot and the remaining tracts, the lowest BIC was obtained under the mixture setting, so we report our results from assuming that gene conversion tract lengths are drawn from the mixture distribution.

For detected tracts with midpoint located within a crossover hotspot, we estimate the mean of the first and second components to be 579.8 bp (95% CI: [574.8, 585.5]) and 20.3 bp (95% CI: [19.7, 21.1]) respectively. We further estimate the mixing proportion for the first component to be 0.0095 (95% CI: [0.00925, 0.01]). We estimate the overall mean to be 25.6 bp (95% CI: [24.9, 26.7]).

For detected tracts with midpoint not located within a crossover hotspot, we estimate the mean of the first and second components to be 813.9 bp (95% CI: [807.7, 819.3]) and 15.5 bp (95% CI: [14.9, 15.6]) respectively. We further estimate the mixing proportion for the first component to be 0.004 (95% CI: [0.00375, 0.004]). We estimate the overall mean to be 18.7 bp (95% CI: [17.9, 18.8]).

## Discussion

By applying the multi-individual IBD method to the UK Biobank whole autosome data, we were able to detect gene conversion events across multiple meioses in the ancestral history of the White British individuals in the UK Biobank [13]. Using this method, 5,961,128 gene conversion tracts were detected, which is several orders of magnitude larger than what had been detected in humans in the past. In the largest pedigree study conducted to detect gene conversions, less than 30,000 gene conversion events were detected from 5,420 trios [11].

We proposed a likelihood-based estimation method to infer the mean gene conversion tract length. Our method is inspired by a previous approach developed by Betran et al., which was applied to gene conversion tracts detected in 34 *Drosophila subobscura* sequences [20]. However, we made several key improvements. First, we define a separate allele conversion probability for each gene conversion tract, based on the density and heterozygosity rate of SNV positions near each tract. Second, we allow gene conversion tract lengths to follow multiple distributions, including a mixture of two geometric components, which has previously been found to appropriately model gene conversion tract lengths in other mammals [9]. Third, we derive the closed-form expression for the distribution of observed tract lengths for each true tract length distribution, which allows for fast and exact calculation of the joint likelihood during maximum likelihood estimation. Finally, we allow for the selection of the best fitting tract length distribution using BIC.

We ran a coalescent simulation incorporating gene conversion events to validate our estimation method. Since we used *msprime* for the simulation, gene conversion tract lengths were necessarily drawn from a geometric distribution. Nonetheless, this simulation allowed us to accurately capture potential biases arising from evolutionary and technical factors such as mutations and genotype errors, as well as methodological factors in the multi-individual IBD detection method used to identify gene conversion tracts [13]. We found that our model accurately estimated the mean gene conversion tract length when the length distribution of gene conversion tracts was correctly specified to be geometric. Our model resulted in biased estimates of the mean gene conversion tract length when the length distribution was incorrectly specified. In most replicates of this simulation study (18 out of 20 replicates), BIC correctly determined the tract length distribution to be geometric.

To further assess the robustness of our model to the misspecification of the tract length distribution, we conducted a separate simulation study where gene conversion tract lengths were drawn from multiple distributions, including a mixture of two geometric components with parameters that were nearly identical to what we estimated using observed tract lengths detected in the UK Biobank whole autosome sequence data. In this study, we found that the model selected by BIC consistently produced relatively unbiased estimates across a range of tract length distributions (see Table 2). Furthermore, when the true tract length distribution was one of the three distributions that we allow for in our model, we found that BIC selects the true distribution in most cases (see Table 3). In this simulation study, our estimate of the mean tract length is slightly upwardly biased (by 7.7 bp) when observed tracts are generated using the mixture distribution with parameters similar to what we inferred in the UK Biobank analysis. However, note that due to the small number of observed tract lengths between 2 bp and 1.5 kb (50), it may be difficult to ascertain the tail of the observed tract length distribution which likely originates from the mixture component with the larger mean. The small number of observed tract lengths is a limitation of this simulation study, but it is difficult to simulate a large number of observed tracts between 2 bp and 1.5 kb when we set the mean gene conversion tract length to be small. This is because simulated gene conversion tracts are unlikely to overlap two heterozygous positions.

Applying our method to nearly one million observed tract lengths detected from the UK Biobank whole autosome data, we found that the mixture setting had the lowest BIC by a large margin. Using this model, we estimated the means of the two geometric distributions to be 16.9 bp (95% CI: [16.4, 17.0]) and 724.7 bp (95% CI: [720.1, 728.7]). The mixing proportion for the geometric distribution with the smaller mean was estimated to be 0.00525 (95% CI: [0.005, 0.00525]). We estimate the overall mean to be 20.6 bp (95% CI: [19.9, 20.7]).

Our estimate of the mean gene conversion tract length is very sensitive to the assumed tract length distribution. When assuming that gene conversion tract lengths are geometric, our model estimates the mean gene conversion tract length to be 459.0 bp (95% CI: [457.3, 460.5]), which is much higher than our estimate under the mixture setting. However, given the large BIC difference between these two models (341,569), we are confident that the mixture distribution is a much better fit to the data. This can also be verified from Fig 3, where we plot the inferred and empirical distributions of observed tract lengths. This result aligns with previous findings in humans [11]. The higher estimate we obtained under the geometric distribution is also consistent with our simulation results. In the simulation assessing the robustness of our method, where we draw gene conversion tract lengths from various distributions, we found that assuming a geometric distribution when the true distribution is a mixture of two geometric components can lead to an inflated estimate of the mean tract length, particularly when one component has a substantially larger mean but contributes relatively few tracts (see Table 2).

We estimated the overall mean gene conversion tract length to be 20.6 bp (95% CI: [19.9, 20.7]), which is shorter than previous estimates. For instance, Palsson et al. reported mean tract lengths of 123 bp (95% CI: [94, 135]) for paternal and 102 bp (95% CI: [71, 125]) for maternal transmissions [11]. Methodological differences between our approach and the NCOurd model used by Palsson et al. may account for this discrepancy [10]. NCOurd requires specifying a penetrance parameter, defined as the probability that a heterozygous position within a gene conversion tract is allele converted. In our framework, we set the allele conversion probability within each tract equal to the local mean heterozygosity rate. This effectively assumes that, for shorter gene conversion tracts (<1.5 kb), all heterozygous positions are allele converted. This would correspond to using a penetrance of one in NCOurd. In contrast, Palsson et al. estimate a fixed penetrance of 0.66 for all detected tracts by using a grid of penetrance values and selecting the one that maximizes the model likelihood. This implies that roughly a third of heterozygous sites within a gene conversion tract do not undergo allele conversion, leading to longer estimated tract lengths. Importantly, penetrance may vary with tract length, making the use of a single penetrance value potentially inappropriate. However, estimating penetrance as a function of the tract length is challenging, especially for short tracts, which often do not overlap with many SNV positions. This limitation has been noted in the original NCOurd publication [10].

Note that we can also vary the penetrance in our method by adjusting the allele conversion probability for each tract (${\hat{\psi }}_{j}$). Specifically, if we denoted the penetrance as ρ, we could use ${\rho \hat{\psi }}_{j}$ as the allele conversion probability for tract j. However, we would need to extend our model fitting procedure to estimate ρ.

There are a few other findings on the length distribution of gene conversion tracts in humans. A sperm-typing study by Jeffreys and May concluded that the mean length is in the range of 55–290 bp [3]. Jeffreys and May inferred the range of mean gene conversion tract lengths (55–290 bp) by comparing observed gene conversion lengths to simulated tracts under geometrically and normally distributed gene conversion tract lengths. However, our simulation where tract lengths are drawn from a mixture distribution suggests that modeling all tracts using a single distribution, without explicitly accounting for outliers, can lead to an inflated estimate of the mean (see Table 2). Using gene conversions detected from HiFi PacBio long-read sequencing of human sperm, and assuming a geometric distribution for tract lengths, Charmouh et al. estimated the mean tract length to be 46 bp (95% CI: [24, 84]), which is closer to our estimate.

Wall et al. analyzed gene conversion tracts shorter than 10 kb in a captive baboon colony using a mixture of two geometric distributions [9]. They estimated that 99.8% of tracts had a mean length of 24 bp (95% CI: [18, 31]), while the remaining tracts had a mean of 4.3 kb (95% CI: [2.6, 4.9]). Both the mixing proportion and the mean of the shorter component are similar to our estimates. Our method is also applicable to other species, but would require population-level sequence data, reliable phasing, and a genetic map for the gene conversion detection.

We ran an additional analysis in which we stratified detected gene conversion tracts from the UK Biobank whole autosome data by whether their midpoints were located within a crossover hotspot. In both sets of tracts, the set of tracts with midpoints located within a crossover hotspot and the remaining tracts, BIC was smallest when assuming a mixture distribution for the true tract length distribution. Comparing the estimated parameters for the mixture distribution in each set, detected tracts with midpoints located within a hotspot were estimated to have a larger proportion of longer tracts (0.0095; 95% CI: [0.00925, 0.01]) compared to the remaining detected tracts (0.004; 95% CI: [0.00375, 0.004]). The mean of the longer component of the mixture distribution was estimated to be smaller for hotspot tracts (579.8 bp; 95% CI: [574.8, 585.5]) compared to the remaining tracts (813.9 bp; 95% CI: [807.7, 819.3]). The mean of the shorter component of the mixture distribution was estimated to be larger for hotspot tracts (20.3 bp; 95% CI: [19.7, 21.1]) compared to the remaining tracts (15.5 bp; 95% CI: [14.9, 15.6]). The overall mean was larger for hotspot tracts (25.6 bp; 95% CI: [24.9, 26.7]) compared to the remaining tracts (18.7 bp; 95% CI: [17.9, 18.8]). These differences in the proportion of longer tracts, and in the mean lengths of the shorter and longer components were significant. This is a preliminary finding and we recommend further analysis to confirm this result. Recombination hotspots correlate with other genomic features such as GC rate [25], so the difference may be caused by factors other than the recombination rate itself.

It is important to acknowledge that our method omits observed tract lengths exceeding 1.5 kb, because we cannot accurately detect observed tract lengths corresponding to longer gene conversion tracts. Complex gene conversion events, which result in both allele converted and non-allele converted heterozygous positions, often span more than 1.5 kb [5]. To appropriately model the lengths of these longer tracts, we would need to apply a detection method that can reliably detect these tracts.

In this study, we did not extend the mixture distribution, which was strongly favored by BIC, to have more than two components. While a mixture model with additional components may better capture the true distribution of gene conversion tract lengths, exploring such models proved computationally challenging due to the complexity of the optimization procedure and the large number of detected gene conversion tracts. Future work may consider more flexible models, such as three-component mixtures, particularly as methods for detecting longer or complex gene conversion events from population-level sequence data become available.

## Supporting information

S1 Appendix The effect of linkage disequilibrium on the distribution of observed tract lengths. (PDF)

S2 Appendix Deriving the marginal distribution of the observed tract length under two alternative settings. (PDF)

S1 FigComparing the CDF of L and the empirical CDF of observed tract lengths detected in the coalescent simulation.We plot the CDF of L truncated between 2 and 1,500 bp (in grey) and the empirical CDF of observed tract lengths between 2 and 1,500 bp detected in the coalescent simulation (in red).(TIF)

S2 FigComparing the CDF of L and the empirical CDF of observed tract lengths generated in the simulation without linkage disequilibrium.We plot the CDF of L truncated between 2 and 1,500 bp (in grey) and the empirical CDF of observed tract lengths between 2 and 1,500 bp generated in the simulation without linkage disequilibrium (in red).(TIF)

S3 Fig Probability distribution functions (log scale) of the ten distributions used to simulate gene conversion tract lengths. We plot the distribution functions of the geometric distribution, the sum of two geometric random variables, the sum of three geometric random variables, the discrete uniform distribution, and the mixture of two geometric components that we draw the gene conversion tract lengths from the simulation study used to assess the robustness of the model.
(TIF)

S4 FigEmpirical distribution of observed tract lengths and model fits in simulation study 1.We plot the inferred distribution of observed tract lengths under our three tract length distributions, and the empirical distribution of observed tract lengths from replicates 7 and 19 of simulation study 1. Probabilities are in the log 10 scale. To obtain the model fits, we average the probability mass at each observed tract length value across estimated allele conversion probabilities for each tract (${\hat{\psi }}_{j}$).(TIF)
