# Supplementary material for: Modeling the length distribution of gene conversion tracts in humans from the UK Biobank sequence data
Source: PLoS Genet. 2025 Nov 17;21(11):e1011951. doi: 10.1371/journal.pgen.1011951 (PMC12643279; doi:10.1371/journal.pgen.1011951)
Supplement: S2 Appendix — (PDF) [file pgen.1011951.s002.pdf]

S2 Appendix: Deriving the marginal distribution of the observed tract length under two alternative settings

We first consider the case in which N is distributed as a sum of two independent and identically distributed geometric random variables each with mean $\phi/2$. We have,

$$P(N = n) = (n-1)\left(1-\frac{2}{\phi}\right)^{n-2}\left(\frac{2}{\phi}\right)^{2}.$$

Letting $\gamma = \frac{2}{\phi}$,

$$P(L = l) = \sum_{n=l}^{\infty} P(L = l|N = n)P(N = n)$$

$$= \begin{cases} \dfrac{\gamma^2(1-\psi)^2}{(\gamma+\psi-\gamma\psi)^2} & \text{if } l = 0 \\ \dfrac{2\gamma^2\psi(1-\psi)}{(\gamma+\psi-\gamma\psi)^3} & \text{if } l = 1 \\ \dfrac{\gamma^2(1-\gamma)^{l-2}\psi^2[(l-3)(\gamma+\psi-\gamma\psi)+2]}{(\gamma+\psi-\gamma\psi)^3} & \text{if } l \geq 2. \end{cases}$$

Then,

$$P(2 \leq L \leq M) = \sum_{l=2}^{M} \frac{\gamma^2(1-\gamma)^{l-2}\psi^2[(l-3)(\gamma+\psi-\gamma\psi)+2]}{(\gamma+\psi-\gamma\psi)^3}$$

$$= \frac{(\gamma+\psi-\gamma\psi)\psi^2[(3-M)\gamma(1-\gamma)^{M-1}-(1-\gamma)^{M-1}-2\gamma+1]+2\gamma\psi^2[1-(1-\gamma)^{M-1}]}{(\gamma+\psi-\gamma\psi)^3}.$$

Then,

$$P(L = l|2 \leq L \leq M) = \frac{P(L = l)}{P(2 \leq L \leq M)}$$

$$= \frac{(\gamma+\psi-\gamma\psi)(l-3)\gamma^2(1-\gamma)^{l-2}+2\gamma^2(1-\gamma)^{l-2}}{(\gamma+\psi-\gamma\psi)[(3-M)\gamma(1-\gamma)^{M-1}-(1-\gamma)^{M-1}-2\gamma+1]+2\gamma[1-(1-\gamma)^{M-1}]}.$$

Similarly to the case where N is geometric, we index our random variable L using j so that L_j represents the random variable corresponding to the observed tract length for detected tract j in our dataset. This time, we also index ψ using j so that an allele conversion happens with probability ψ_j at every position within the jth detected tract (the estimation of ψ_j is described in the section, Estimating the allele conversion probability for each detected tract). We have,

$$P\left(L_j = l_j \middle| 2 \leq L_j \leq M\right)$$
$$= \frac{\left(\gamma + \psi_j - \gamma\psi_j\right)\left(l_j - 3\right)\gamma^2(1-\gamma)^{l_j-2} + 2\gamma^2(1-\gamma)^{l_j-2}}{\left(\gamma + \psi_j - \gamma\psi_j\right)[(3-M)\gamma(1-\gamma)^{M-1} - (1-\gamma)^{M-1} - 2\gamma + 1] + 2\gamma[1-(1-\gamma)^{M-1}]}.$$

We next consider the case where N is distributed as a mixture of two geometric components. We let the two geometric means be ϕ_1 and ϕ_2, and let w_1 represent the mixing proportion of the first component. We have,

$$P(N = n) = w_1\left(1 - \frac{1}{\phi_1}\right)^{n-1}\frac{1}{\phi_1} + (1 - w_1)\left(1 - \frac{1}{\phi_2}\right)^{n-1}\frac{1}{\phi_2}.$$

Letting $\lambda_1 = 1/\phi_1$ and $\lambda_2 = 1/\phi_2$,

$$P(L = \ell) = \sum_{n=\ell}^{\infty} P(L = \ell | N = n)P(N = n)$$

$$= \begin{cases} \dfrac{w_1\lambda_1(1-\psi)}{\lambda_1 + \psi - \lambda_1\psi} + \dfrac{(1-w_1)\lambda_2(1-\psi)}{\lambda_2 + \psi - \lambda_2\psi} & \text{if } \ell = 0 \\ \dfrac{w_1\lambda_1\psi}{(\lambda_1 + \psi - \lambda_1\psi)^2} + \dfrac{(1-w_1)\lambda_2\psi}{(\lambda_2 + \psi - \lambda_2\psi)^2} & \text{if } \ell = 1 \\ \dfrac{w_1\lambda_1\,(1-\lambda_1)^{\ell-1}\,\psi^2}{(\lambda_1 + \psi - \lambda_1\psi)^2} + \dfrac{(1-w_1)\lambda_2(1-\lambda_2)^{\ell-1}\,\psi^2}{(\lambda_2 + \psi - \lambda_2\psi)^2} & \text{if } \ell \geq 2. \end{cases}$$

Then,

$$P(2 \le L \le M) = \sum_{l=2}^{M} \left[\frac{w_1 \lambda_1 \, (1-\lambda_1)^{\ell-1} \, \psi^2}{(\lambda_1 + \psi - \lambda_1 \psi)^2} + \frac{(1-w_1)\lambda_2 (1-\lambda_2)^{\ell-1} \, \psi^2}{(\lambda_2 + \psi - \lambda_2 \psi)^2} \right]$$

$$= \frac{w_1 \psi^2 [(1-\lambda_1) - (1-\lambda_1)^M]}{(\lambda_1 + \psi - \lambda_1 \psi)^2} + \frac{(1-w_1)\psi^2 [(1-\lambda_2) - (1-\lambda_2)^M]}{(\lambda_2 + \psi - \lambda_2 \psi)^2}.$$

Then,

$$P(L = \ell | 2 \le L \le M) = \frac{P(L = \ell)}{P(2 \le L \le M)}$$

$$= \frac{\frac{w_1 \lambda_1 \, (1-\lambda_1)^{\ell-1} \, \psi^2}{(\lambda_1 + \psi - \lambda_1 \psi)^2} + \frac{(1-w_1)\lambda_2 (1-\lambda_2)^{\ell-1} \, \psi^2}{(\lambda_2 + \psi - \lambda_2 \psi)^2}}{\frac{w_1 \psi^2 [(1-\lambda_1) - (1-\lambda_1)^M]}{(\lambda_1 + \psi - \lambda_1 \psi)^2} + \frac{(1-w_1)\psi^2 [(1-\lambda_2) - (1-\lambda_2)^M]}{(\lambda_2 + \psi - \lambda_2 \psi)^2}}.$$

Again using j to index detected tracts,

$$P\left(L_j = \ell_j \middle| 2 \le L_j \le M\right) = \frac{\frac{w_1 \lambda_1 \, (1-\lambda_1)^{\ell_j-1} \, \psi_j^2}{\left(\lambda_1 + \psi_j - \lambda_1 \psi_j\right)^2} + \frac{(1-w_1)\lambda_2 (1-\lambda_2)^{\ell_j-1} \, \psi_j^2}{\left(\lambda_2 + \psi_j - \lambda_2 \psi_j\right)^2}}{\frac{w_1 {\psi_j}^2 [(1-\lambda_1) - (1-\lambda_1)^M]}{\left(\lambda_1 + \psi_j - \lambda_1 \psi_j\right)^2} + \frac{(1-w_1)\psi_j^2 [(1-\lambda_2) - (1-\lambda_2)^M]}{\left(\lambda_2 + \psi_j - \lambda_2 \psi_j\right)^2}}.$$

In practice, we plug in $M = 1500$ because we exclude all observed tract lengths longer than 1500 bp detected from the UK Biobank whole autosome data.
