# Supplementary figures and images for: Modeling the length distribution of gene conversion tracts in humans from the UK Biobank sequence data
Source: PLoS Genet. 2025 Nov 17;21(11):e1011951. doi: 10.1371/journal.pgen.1011951 (PMC12643279; doi:10.1371/journal.pgen.1011951)

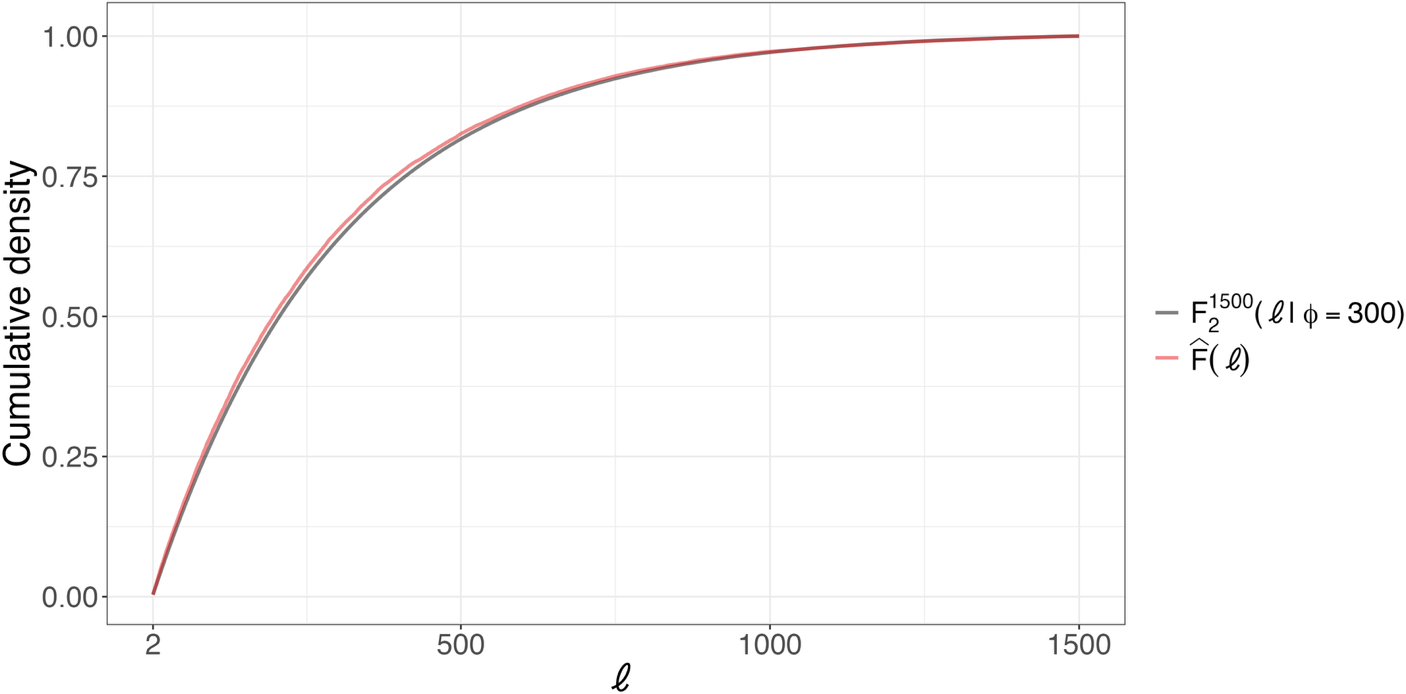

Supplement: S1 Fig — We plot the CDF of L truncated between 2 and 1,500 bp (in grey) and the empirical CDF of observed tract lengths between 2 and 1,500 bp detected in the coalescent simulation (in red). (TIF) [file pgen.1011951.s003.tif]

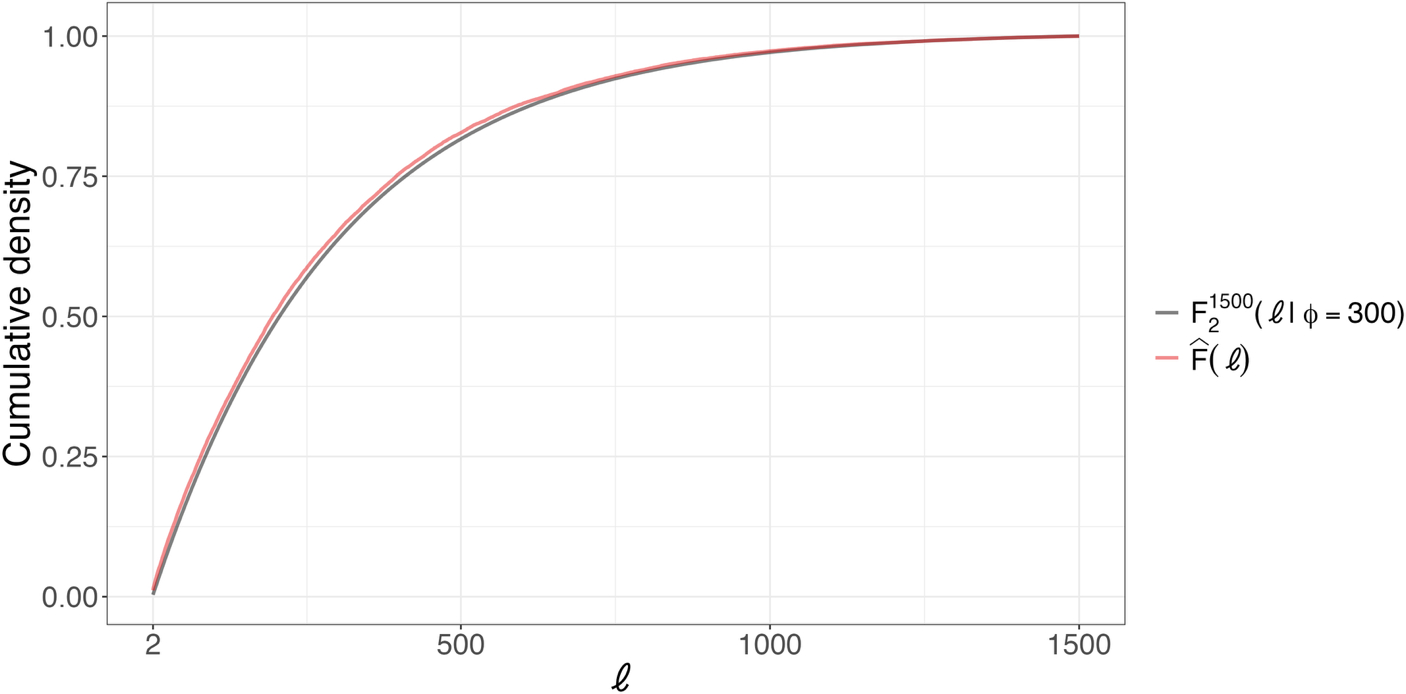

Supplement: S2 Fig — We plot the CDF of L truncated between 2 and 1,500 bp (in grey) and the empirical CDF of observed tract lengths between 2 and 1,500 bp generated in the simulation without linkage disequilibrium (in red). (TIF) [file pgen.1011951.s004.tif]

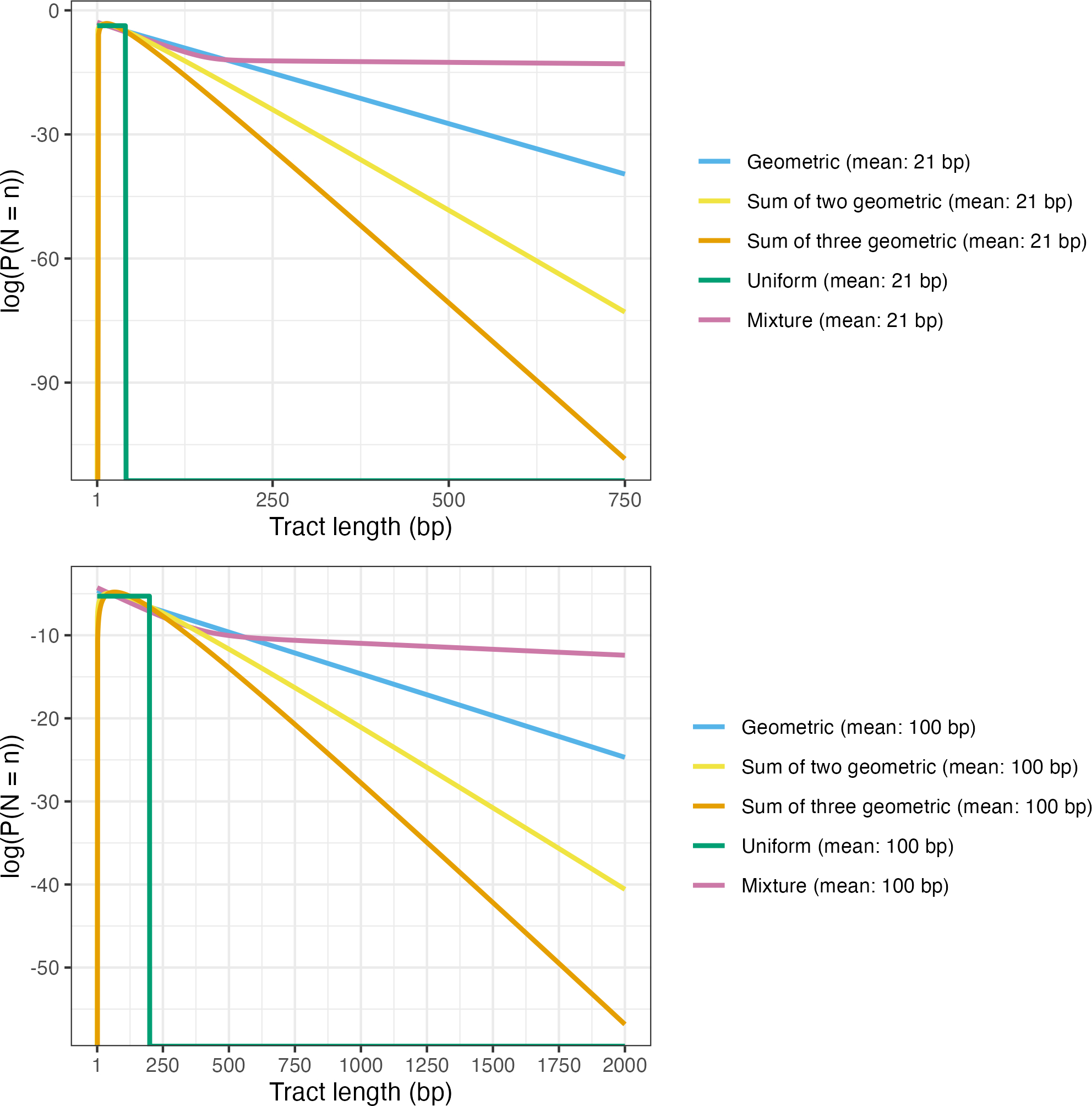

Supplement: S3 Fig — We plot the distribution functions of the geometric distribution, the sum of two geometric random variables, the sum of three geometric random variables, the discrete uniform distribution, and the mixture of two geometric components that we draw the gene conversion tract lengths from the simulation study used to assess the robustness of the model. (TIF) [file pgen.1011951.s005.tif]

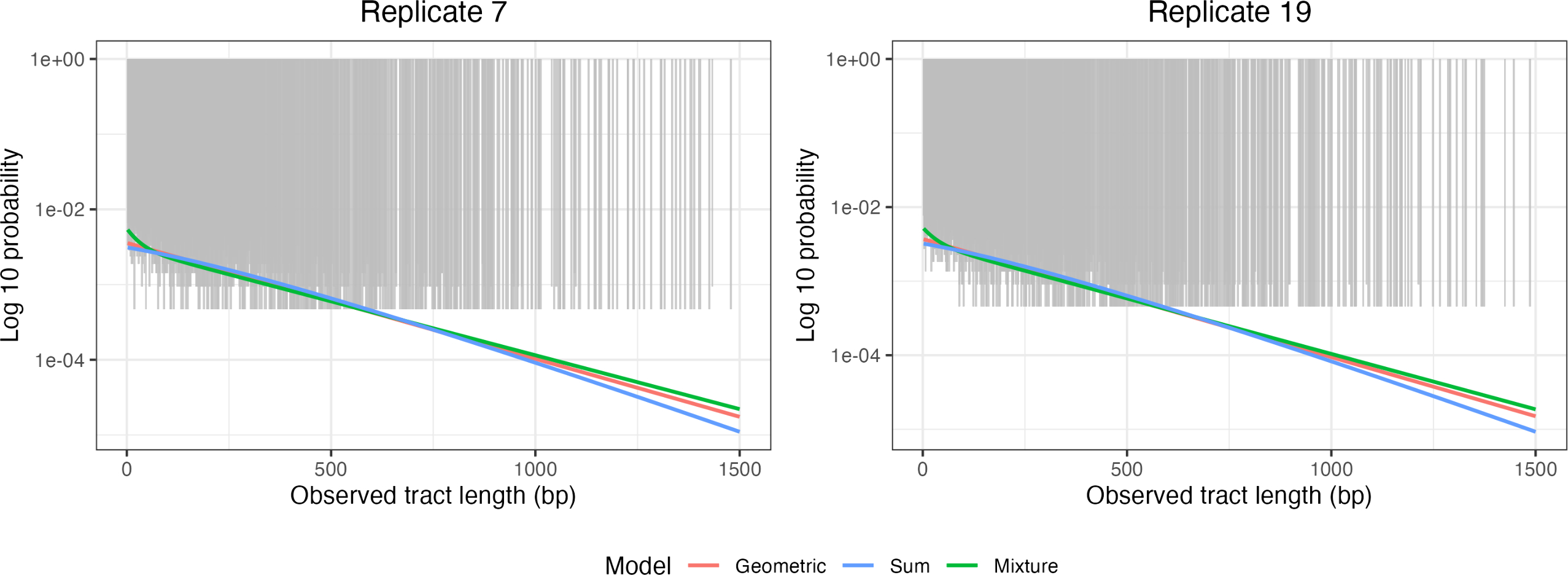

Supplement: S4 Fig — We plot the inferred distribution of observed tract lengths under our three tract length distributions, and the empirical distribution of observed tract lengths from replicates 7 and 19 of simulation study 1. Probabilities are in the log 10 scale. To obtain the model fits, we average the probability mass at each observed tract length value across estimated allele conversion probabilities for each tract (ψ^j). (TIF) [file pgen.1011951.s006.tif]
